# 

**Published:** 1946

**Authors:** 


### The Medical Library of the University of Bristol

WITH WHICH IS INCORPORATED

**The Library of the Bristol Medico-Chirurgical Society**

---

*The following donations have been received since the publication of the list in the last issue.*

| | |
|---|---|
| Professor R. J. Brocklehurst (1) ... ... ... | 4 Volumes. |
| Brompton Hospital (2) ... ... ... ... ... | 1 Volume. |
| J. Lacy Firth, Esq. (3) ... ... ... ... ... | 34 Volumes. |
| Henry Phipps Institute (4) ... ... ... ... | 1 Volume. |
| Dr. G. D. Kersley (5) ... ... ... ... ... | 1 Volume. |
| Masson et Cie., Paris (6) ... ... ... ... | 2 Volumes. |
| H. E. Matthews, Esq. (7) ... ... ... ... | 79 Volumes. |
| Michell Clarke Memorial Fund (8) ... ... ... | 16 Volumes. |
| W. B. Saunders Co. (9) ... ... ... ... ... | 1 Volume. |
| J. E. Shaw Bequest (10) ... ... ... ... | 2 Volumes. |
| A. Rendle Short, Esq. (11) ... ... ... ... | 1 Volume. |
| Munro Smith Fund (12) ... ... ... ... ... | 1 Volume. |
| Dr. J. O. Symes (13) ... ... ... ... ... | 1 Volume. |
| A. J. M. Wright, Esq. (14) ... ... ... ... | 20 Volumes. |
| John Wright & Sons Ltd. (15) ... ... ... | 1 Volume. |

Unbound periodicals have also been received from Professor C. Bruce Perry, Dr. S. B. Adams, J. C. Shipham, Esq., and Messrs. John Wright and Sons Ltd.

### THE ONE HUNDRED AND EIGHTY-SECOND LIST OF BOOKS.

The figures in round brackets refer to the figures after the names of the donors. The books to which no such figures have been attached have either been bought from the Library Fund or received through the *Journal*.

| | | | |
|---|---|---|---|
| **Abramson, D. I.** .. | *Vascular Responses in the Extremities of Man* | (7) | 1946 |
| **Adriani, J.** .. .. .. .. | *Pharmacology of Anesthetic Drugs* | .. 2nd Ed. | 1946 |
| **Bailey, H. H.** .. .. | *Physical Signs in Clinical Surgery* | ..10th Ed. | 1946 |
| **Bailey, H. H.** .. .. | *Surgery of Modern Warfare* .. .. .. | .. (11) | 1941 |
| **Bailey, H. H., and Love, R. J. M.** | *Short Practice of Surgery* | .. 7th Ed. | 1946 |

| | | | |
|---|---|---|---|
| **Ballenger, W. L., and Ballenger, H. C.** | *Diseases of the Nose, Throat and Ear* | 8th Ed. (8) | 1946 |
| **Bertwistle, A. P.** | *Descriptive Atlas of Radiographs* | 6th Ed. | 1946 |
| **Bishop, P. M. F.** | *Gynaecological Endocrinology* | (8) | 1946 |
| **Braithwaite, J. U.** | *Infant Feeding in General Practice* | 2nd Ed. | 1943 |
| **British Medical Association** | *A Charter for Health* | | 1946 |
| **Brock, R. C.** | *Anatomy of the Bronchial Tree* | | 1946 |
| **Brödel, Max** | *Three Unpublished Drawings of the Anatomy of the Human Ear* | (9) | 1946 |
| **Cameron, H. C.** | *Nervous Child* | 5th Ed. | 1946 |
| **Clark, W. E. le G.** | *Practical Anatomy* | | 1946 |
| **Conybeare, J. (ed.)** | *Text-book of Medicine* | 8th Ed. (8) | 1946 |
| **Crosse, V. M.** | *Premature Baby* | | 1946 |
| **Cruickshank, E. W. H.** | *Food and Nutrition* | | 1946 |
| **Curran, D., and Guttman, E.** | *Psychological Medicine* | 2nd Ed. | 1946 |
| **Dandy, W. E.** | *Intracranial Arterial Aneurysms* | (8) | 1945 |
| **Davis, L.** | *Principles of Neurological Surgery* | 3rd Ed. | 1946 |
| **Delay, J.** | *L'électro-choc et la Psycho-physiologie* | (6) | 1946 |
| **Dubos, R. J.** | *Bacterial Cell* | | 1945 |
| **Duke-Elder, S.** | *Practice of Refraction* | 4th Ed. | 1946 |
| **Dunlop, D. M., and others.** | *Text-book of Medical Treatment* | 4th Ed. | 1946 |
| **East, T.** | *Cardiovascular Disease in General Practice* | 2nd Ed. | 1946 |
| **Farrell, J. T.** | *Rontgen Diagnosis of Gastrointestinal Tract* | | 1946 |
| **Fearon, W. R.** | *Introduction to Bio-chemistry* | 3rd Ed. (8) | 1946 |
| **Fiessinger, N.** | *Clinique et Investigations* | (6) | 1946 |
| **Fisher, R. A.** | *Statistical Methods for Research Workers* | 10th Ed. | 1946 |
| **Frazer, W. M., and Stallybrass, C. O.** | *Text-book of Public Health* | 11th Ed. | 1946 |
| **Glaister, J.** | *Medical Jurisprudence and Toxicology* | 8th Ed. (8) | 1945 |
| **Gray, H.** | *Anatomy* | 17th Ed. (13) | 1909 |
| **Gray, H.** | *Anatomy* | 29th Ed. | 1946 |
| **Gullan, M. A.** | *Theory and Practice of Nursing* | 5th Ed. | 1946 |
| **Gustafson, G.** | *Structure of Human Dental Enamel* | | 1945 |
| **Guthrie, D.** | *History of Medicine* | (12) | 1945 |
| **Hardy, G. H.** | *Book of the Fly* | (1) | 1915 |
| **Harvey, W. C., and Hill, H.** | *Milk: Production and Control* | 2nd Ed. | 1946 |
| **Henry, S. A.** | *Cancer of the Scrotum* | | 1946 |
| **Hogeboom, F. E.** | *Practical Pedodontia* | 4th Ed. | 1938 |
| **Howell, W. H.** | *Text-book of Physiology.* 15th Ed. by J. F. Fulton | | 1946 |
| **Kersley, G. D.** | *Rheumatic Diseases* | 2nd Ed. (5) | 1945 |
| **Kraus, F. (ed.)** | *Sparsame Sachgeniässe Krankenbehandlung* | 2nd Ed. (1) | 1927 |
| **Lawrence, R. D.** | *Diabetic Life* | 13th Ed. | 1945 |
| **Levine, S. A.** | *Clinical Heart Disease* | 3rd Ed. (8) | 1946 |
| **Lewis, Sir T.** | *Diseases of the Heart* | 4th Ed. (8) | 1946 |
| **Lewis, Sir T.** | *Pain* | | 1946 |
| **Lewis, Sir T.** | *Vascular Disorders of the Limbs* | 2nd Ed. (8) | 1946 |

**Lishman, F. J. G.** .. *Handbook for Assistant Medical Officers of Health* 2nd Ed. 1946

**Lull, C. B., and Hingson, R. A.** *Control of Pain in Childbirth* 2nd Ed. (8) 1945

**McElligott, M.** .. .. *Spanish–English Medical Dictionary* .. .. .. 1946

**McGehee, W. H. O.** .. *Text-book of Operative Dentistry* .. 2nd Ed. 1945

**MacIntosh, R. R., and Mushin, W. W.** *Local Anaesthesia : Brachial Plexus* (8) 1944

**MacIntosh, R. R., and Mushin, W. W.** *Physics for the Anaesthetist* .. (8) 1946

**Marquand, H. S. le, and Tozer, F. H. W.** *Endocrine Disorders in Childhood and Adolescence* .. .. .. .. .. .. .. .. 1943

**Martindale, W.** .. .. *Extra Pharmacopoeia.* Vol. 2 .. .. 22nd Ed. 1943

**May, C. H., and Worth, C.** *Manual of Diseases of the Eye* .. 9th Ed. (8) 1946

**Micks, R. H.** .. .. *Essentials of Materia Medica, Pharmacology, and Therapeutics* .. .. .. .. .. .. .. 3rd Ed. 1944

**Patton, W. S., and Evans, A. M.** *Insects, Ticks, and Venomous Animals* 2 vols. (10) 1929–31

*Penicillin : Its Properties, Uses, and Preparations* 1946

**Prince, M.** .. .. .. *Dissociation of a Personality* .. .. .. (1) 1919

**Ranson, S. W.** .. .. *Anatomy of the Nervous System* .. .. 7th Ed. 1943

**Rogers, L., and Megaw, J. W. D.** *Tropical Medicine* .. .. .. 5th Ed. 1946

**Rogers, L., and Muir, E.** *Leprosy* .. .. .. .. .. .. .. .. 3rd Ed. 1946

**Romanis, W. H. C., and Mitchiner, P. H.** *Science and Practice of Surgery* 7th Ed., 2 vols. 1944

**Schall, W. E.** .. .. *X-rays* .. .. .. .. .. .. .. .. 5th Ed. (15) 1946

**Sheldon, W.** .. .. .. *Diseases of Infancy and Childhood* 5th Ed. (8) 1946

**Smith, H. W.** .. .. *Lectures on the Kidney* .. .. .. .. .. .. (8) 1943

**Smith, J. C.** .. .. .. *Chemistry and Metallurgy of Dental Materials* .. 1946

**Soffer, L. J.** .. .. .. *Diseases of the Adrenals* .. .. .. .. .. .. 1946

**Stone, M. J., and Dufault, P.** *Diagnosis and Treatment of Pulmonary Tuberculosis* .. .. .. .. .. .. .. .. .. .. 1946

**Thorpe, W. V.** .. .. *Bio-chemistry for Medical Students* .. 3rd Ed. 1946

**Traquair, H. M.** .. .. *Introduction to Clinical Perimetry* .. .. 5th Ed. 1946

**True, W. P.** .. .. .. *First Hundred Years of the Smithsonian Institution, 1846–1946* .. .. .. .. .. .. .. .. .. .. .. 1946

**Walker, K. M., and Walker, E. M.** *On Being a Father* .. .. .. (1) 1928

**Weinbren, M.** .. .. *Manual of Toniography* .. .. .. .. .. .. .. .. 1946

**Widdowson, T. W.** .. *Special or Dental Anatomy and Physiology and Dental Histology* .. .. .. 7th Ed., 2 vols. 1946

**Wilson, J. V.** .. .. *Pathology of Traumatic Injury* .. .. .. .. .. 1946

**Woolmer, R.** .. .. .. *Anaesthetics Afloat* .. .. .. .. .. .. .. .. .. .. 1942

**Wyburn-Mason, R.** .. *Vascular Abnormalities and Tumours of the Spinal Cord* .. .. .. .. .. .. .. .. .. .. 1943

**Yellowlees, H.** .. .. *The Human Approach* .. .. .. .. .. .. .. .. 1946

TRANSACTIONS, REPORTS, JOURNALS, ETC.

Brompton Hospital Reports. Vol. 14 .. .. .. .. .. .. .. .. .. .. (2) 1945

Collected Papers of the Mayo Clinic. Vol. 37, 1945 .. .. .. .. .. .. 1946

Radiology : Cumulative Index. Vols. 1–39. 1923–1942 .. .. .. .. .. 1943

Report of Henry Phipps Institute, 1942–43 .. .. .. .. .. .. .. .. (4) 1944

Year-book of Pharmacy, 1870–1945 .. .. .. .. .. .. .. .. .. .. .. (7)

### Publications Received

From MESSRS. JOHN WRIGHT & SONS LTD.:

*Synopsis of Surgical Anatomy.* By A. LEE MCGREGOR. Sixth Edition. 1946. Price 25s.

*A Short Handbook of Practical Anaesthetics.* By HÖEL PARRY-PRICE. 1946. Price 12s. 6d.

*Eye Surgery.* By H. B. STALLARD. 1946. Price 50s.

*British Journal of Surgery.* Vol. 34, No. 134. October, 1946. Price 12s. 6d.

From MESSRS. WILLIAM HEINEMANN MEDICAL BOOKS LTD.:

*Text-book of Gynaecology.* By J. H. PEEL. Second Edition. 1946. Price 21s.

From MESSRS. HENRY SCHUMANN, NEW YORK:

*The Centennial of Surgical Anesthesia.* Compiled by JOHN F. FULTON and MADELINE E. STANTON. 1946. Price $4.00.

*A Memoir on a New Use of Sulphuric Ether.* By W. T. G. MORTON. 1847. With a foreword by JOHN F. FULTON. 1946. Price $1.50.

From CHRISTOPHER JOHNSON PUBLISHERS LTD.:

*The Renaissance and Its Influence on English Medicine, Surgery and Public Health* (Thomas Vicary Lecture, 1945). By Sir ARTHUR S. MACNALTY. 1946. Price 5s.

### Local Medical Notes

**New Year Honours.**—Surgeon Rear Admiral H. R. B. Hull, R.N., has been awarded the C.B.

ON Wednesday, October 23rd, the South-Western Division of the Royal Medico-Psychological Association held a meeting at the Bristol Mental Hospital, Fishponds, by kind permission of the Visiting Committee. A number of medical guests were invited, including all members of the Faculty of the Bristol Royal Hospital. They were able to visit the new Research Laboratories, and afterwards were entertained to luncheon. The new laboratories have been designed to investigate certain physiological, biochemical and endocrinological aspects of neuroses and psychoses. The unit is not yet complete, but the main Biochemical Laboratory, animal house, and part of the Biological Department were in use.

The guests saw many pieces of special apparatus in use, including those for investigating metabolism and blood iodine, estimation of keto-steroids, and the oxygen consumption of infantile and adult rats. They also saw hypophysectomy and adrenalectomy of rats. At the close of the demonstration, there was an exhibition of pictures and objects representative of psychotic and neurotic art. As many members as possible stayed for a meeting in the afternoon, to hear a discussion of the research programme and some of the problems of establishing physiological correlates of mental behaviour.

EXAMINATION RESULTS.

Examination passed:—

**F.R.C.S., Edinburgh.**—H. E. Pearse, M.D., Bristol.

**University of Bristol.**—Students of the University have recently passed the following examinations:—

M.B., CH.B.—*Final Examination:* J. H. Beatson, P. J. Chapman, Sheila E. Fraser, N. G. Glen, J. C. Little, Dorothy M. Reece, R. Simpson-White, Kathleen G. Wright. *In Group II, completing the Examination:* J. L. Dunscombe, D. Purnell, R. G. G. Taylor.

M.B., CH.B.—*Final Examination (Section I):* Margaret J. Andrews, G. F. Bigwood, F. G. Bolton, D. H. Bowden, W. L. Codd, D. M. Cunningham, R. S. G. Davies, W. J. Gall, N. M. Gibbs, C. E. Halliday, Pamela H. L. Hamilton, G. J. S. Herdman, Stella C. Hill, J. A. G. Holt, S. F. Iles, Una P. Jones, A. H. Laxton, Lisa E. Mandelbaum (*a*), C. A. Mills, G. E. Mitchelmore, Jean E. Morrell, J. P. Norris, G. G. Portch (*a*), E. Saphier, P. W. Seargeant (*b*), M. H. Weiss.

M.B., CH.B.—*Second Examination (Section II):* R. M. Barnes. *In Elementary Pathology, completing the Examination:* (Mrs.) Dorothy Field.

B.D.S.—*Final Examination. In Section II (Dental Surgery), completing the Examination:* E. J. R. Morgan, P. J. Sheldrick. *In Section I only:* R. J. Brownlee, A. J. P. Cousins.

B.D.S.—*Third Examination. In Dental Mechanics and the Properties of Materials used in Dental Practice, completing the Examination:* P. P. Griffiths, A. R. Owen.

L.D.S.—*Final Examination. In Section II (Dental Surgery), completing the Examination:* A. D. Baxter, P. J. Blyth, J. P. Rees. *In Section I only:* T. C. Hughes.

L.D.S.—*Third Examination. In Dental Mechanics and The Properties of Materials used in Dental Practice, completing the Examination:* D. Clifford, L. B. Howell, H. T. Ivory.

D.P.H.—*Preliminary Examination:* R. G. Brennan, Joyce W. Brown, Marjorie Bryan, A. Donaldson, Clara J. Fraser, A. T. Hunt, S. Ludkin, D. G. MacNeill, N. R. Matheson, Marjorie Organ, Joan Williams.

D.P.M.—*Part I:* G. M. Gibb, E. C. Kite, J. McGhie, J. F. D. Murphy. *In Psychology only:* W. L. Jones. *In Anatomy and Physiology of the Nervous System only:* Lorna M. Brierley, H. E. D. Flack, W.. A. Heaton-Ward, R. Maggs, H. J. L. O'Sullivan, H. A. Sutherland.

(*a*) With Distinction in Materia Medica, Pharmacy, Pharmacology, etc.

(*b*) With Distinction in Materia Medica, Pharmacy, Pharmacology, etc., and in Pathology and Bacteriology.

### Index to Vol. LXIII

Agnew, K. M.—British Red Cross in N.W. Europe, 31

Appointments, 55, 121

Arthritis.—G. D. Kersley, 11

Beck, D. J. K.—Tumours of the Central Nervous System, 65

Book Reviews, 43, 84, 119, 148

British Red Cross in N.W. Europe.—Lieut.-Col. K. M. Agnew, 31

Cairns, H.—Tumours of the Central Nervous System, 73

Campaigning Notes.—Col. M. Herford, 136

Carleton, H. H.—Presidential Address: "Nature and Science," 1.

Dioscorides Phakas.—M. Nierenstein, 75

Editorial Notes:
Sir Philip Robert Morris, 47
University of Bristol £300,000 Extension Scheme, 150

English Doctors from Smollett to Trollope.—XXXIII Long Fox Memorial Lecture.—E. Watson-Williams, 89

Hemphill, R. E.—Pituitary Cachexia, 116

Herapath, C. E. K.—Obituary, 152

Herford M.—Campaigning Notes, 136

Hyperpiesia in Dockers.—J. M. Naish, 106

Iron in Blood, Urine and Sputum, Dioscorides Phakas.—M. Nierenstein, 75

Kersley, G. D.—Rheumatoid Arthritis, 11

Library Notes, 50, 86, 157

Long Fox Memorial Lectures.—XXXIII, E. Watson-Williams, English Doctors from Smollett to Trollope, 89; XXXIV, M. Skene, Plant Hormones, 125

Moore, R. H., Injection of Pentothal, Inhalation of Food, 82

Morris, Sir Philip (Ed. Note), 47

Naish, J. M.—Hyperpiesia in Dockers, 106

"Nature and Science" (Presidential Address).—H. H. Carleton, 1

Nervous System, Central and Tumours.—H. Cairns, 73

Nierenstein, M.—Iron in Blood, Urine and Sputum, Dioscorides Phakas, 75

"Night-Nurse's Paralysis."—G. de M. Rudolf, 132

Obituary.—C. E. K. Herapath, 152

Pentothal Injection.—R. H. Moore, 82

Peptic Ulcer.—N. C. Tanner, 16

Pituitary Cachexia.—R. E. Hemphill, 116

Plant Hormones, XXXIV Long Fox Memorial Lecture.—M. Skene, 125

Postgraduate Studies.—122

Presidential Address: "Nature and Science."—H. H. Carleton, 1

Rheumatoid Arthritis.—G. D. Kersley, 11

Rudolf, G. de M.—"Night-Nurse's Paralysis," 132

Skene, M., XXXIV Long Fox Memorial Lecture.—Plant Hormones, 125

Tanner, N. C.—Peptic Ulcer, 16

Tumours of the Central Nervous System.—D. J. K. Beck, 65; H. Cairns, 73

University Extension Scheme, 150

(University) Postgraduate Studies, 122

Watson-Williams, E., XXXIII Long Fox Memorial Lecture.—English Doctors from Smollett to Trollope, 89

